# Structural Determinant and Its Underlying Molecular Mechanism of STPC2 Related to Anti-Angiogenic Activity

**DOI:** 10.3390/md15020048

**Published:** 2017-02-21

**Authors:** Min Hu, Ning Cui, Zhixiang Bo, Feixiang Xiang

**Affiliations:** 1 Department of Cardiovascular Surgery, Tongji Hospital, Tongji Medical College, Huazhong University of Science and Technology, Wuhan 430030, China; minhstpc1203@gmail.com (M.H.); zhixiangbstpc1203@yahoo.com (Z.B.); 2 Department of Gastroenterology, Renmin Hospital of Wuhan University, Wuhan 430060, China; ncuistpc1203@gmail.com; 3 Department of Ultrasound, Union Hospital, Tongji Medical College, Huazhong University of Science and Technology, Wuhan 430022, China

**Keywords:** *Sargassum thunbergii*, desulfated, anti-angiogenesis, VEGFR2

## Abstract

In this study, we aimed to use different strategies to further uncover the anti-angiogenic molecular mechanism of a fucoidan-like polysaccharide STPC2, isolated from brown alga *Sargassum thunbergii*. A desulfated derivative, STPC2-DeS, was successfully prepared and identified. The native polysaccharide and desulfated product were subjected to evaluate their anti-angiogenic effects. In the tube formation assay, STPC2 showed dose-dependent inhibition. In addition, STPC2 could distinctly inhibit the permeation of HUVEC cells into the lower chamber. Moreover, a significant reduction of microvessel density was observed in chick chorioallantoic membrane assay treated with STPC2. Meanwhile, STPC2 was found to repress the VEGF-induced neovessel formation in the matrigel plug assay in vivo. However, STPC2-DeS failed to suppress the anti-angiogenic activity via these in vitro and in vivo strategies. In addition, we demonstrated that STPC2 could significantly downregulate the phosphorylation of VEGFR2 and its related downstream Src family kinase, focal adhesion kinase, and AKT kinase. Furthermore, surface plasmon resonance assay revealed that STPC2 bound strongly to VEGF to interfere with VEGF–VEGFR2 interaction. Taken together, these results evidently demonstrated that STPC2 exhibited a potent anti-angiogenic activity through binding to VEGF via sulfated groups to impede VEGF–VEGFR2 interaction, thus affected the downstream signaling molecules.

## 1. Introduction

Angiogenesis, regarded as a process of forming new blood vessels, is essential for tissue repair and organ growth. However, an imbalance in the process is also highly related to numerous malignant, inflammatory, ischemic, and infectious disorders [1,2]. A number of signaling networks have been studied in the regulation of angiogenesis, including PI3K/AKT signaling and RAS/MAPK signaling [3,4,5]. These signalings are activated by growth factors or angiogenesis inducers such as fibroblast growth factor (FGF) and vascular endothelial growth factor (VEGF) through binding to their corresponding receptors. This binding initiates the phosphorylation of receptors, resulting in the activation of downstream signaling pathways and inducing the VEGF and HIF-1 expression, thereby stimulating the angiogenesis [5,6]. Thus, negative regulation of the interaction between growth factors and receptors may be an effective therapy strategy for the modulation of neovascularization.

Previously, the highly sulfated polysaccharides heparin and heparan sulfate extracted from animal tissues have been demonstrated to be the most potent anti-angiogenic agents due to an efficient binding to VEGF and FGF1 [7]. Intriguingly, fucoidans, a set of sulfated polysaccharides extracted from brown algae, attract more attention for its effective regulation of angiogenesis. In a previous report, a fucoidan-like polysaccharide STPC2 was isolated from a brown alga *Sargassum thunbergii*. STPC2 could significantly inhibited tube formation and migration of human umbilical vein vascular endothelial cells (HUVECs), but the underlying mechanism of anti-angiogenesis has not been elucidated well. In this study, we aimed to demonstrate its structural determinants and the underlying mechanism for its regulation on angiogenesis.

## 2. Results

### 2.1. Preparation of Desulfated Derivatives

**Figure 1:**
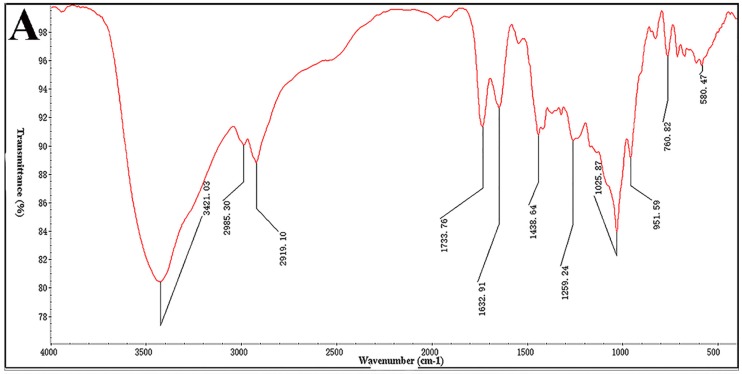

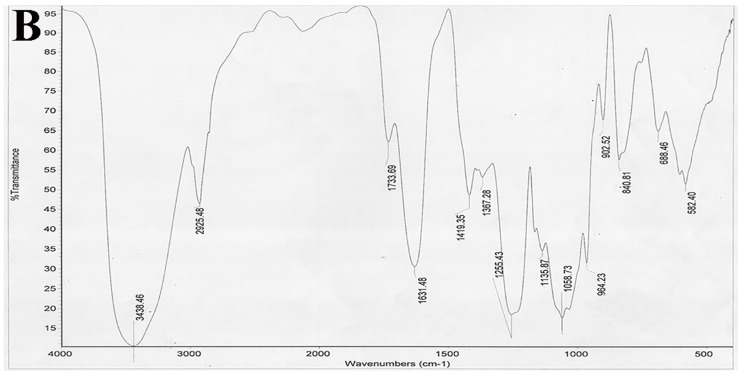
(**A**) Infra-red (IR) spectrum of desulfated derivative STPC2-DeS; and (**B**) the IR spectrum of STPC2.

In the previous study, a fucoidan-like polysaccharide was isolated from boiling-water extract of *Sargassum thunbergii*. It was found that STPC2 was composed of a high ratio of fucose. In addition, its sulfate content was also relatively high, up to 27.8% [8]. In previous studies and reviews, the sulfation was reported to be associated with the contribution of anti-angiogenesis activity [7,9]. To further illustrate whether the sulfation of STPC2 contributed to its anti-angiogenesis activity, we applied the chemical desulfation method to STPC2. As the method employed in these reports [10,11], STPC2 was subjected to H^+^ column and neutralized with pyridine, and re-dissolved in Me_2_SO-MeOH mixture to remove the sulfate group. The remove of sulfate was monitored by the disappearance of the S=O band at 1259.24 cm^−1^ in the spectrum as shown in Figure 1A. Compared to the IR of STPC2, the absorption at 1255.43 cm^−1^ (Figure 1B) disappeared, suggesting that the process of desulfation was successful. In addition, the sulfur content of STPC2-DeS was determined to be 0.8% by elemental analysis, indicating that almost 91.4% of sulfate groups had been removed. Subsequently, its sugar compositions were measured to contain fucose, xylose, galactose, and glucuronic acid, in a ratio of 7.8:3.2:1.9:1.0. Compared to its native polysaccharide constituents, the desulfated products did not show a significant change of the sugar composition. Moreover, we determined its weight-average molar mass (*M*_w_) again. The HPGPC results indicated that the *M*_w_ of STPC2-DeS was 44.5 kD. The slight reduction in the molecular weight was in accordance with the loss of the sulfate group.

### 2.2. Effect of Sulfation in STPC2 on Its Anti-Angiogenesis In Vitro and In Vivo

**Figure 2:**
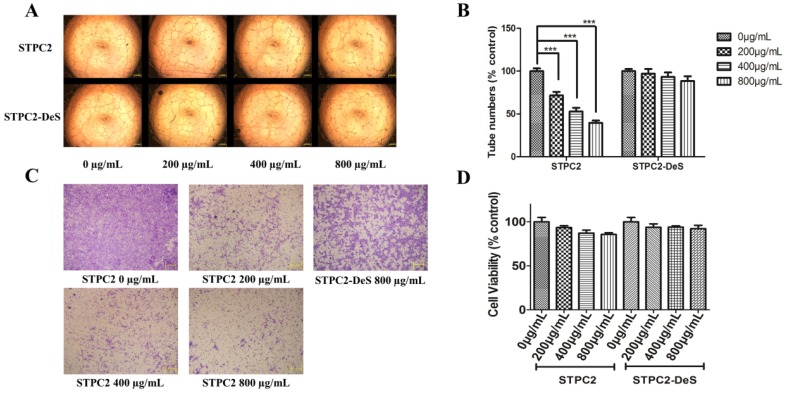
STPC2 inhibited angiogenesis through tube formation assay and cell migration transwell assay in vitro. (**A**) Tube formation of HUVEC cells on matrigel. Cells were treated with STPC2 or STPC2-DeS at the concentrations of 0, 200, 400, and 800 µg/mL for 24 h; (**B**) Quantification of tube numbers in the treatment of STPC2 or STPC2-DeS. All data were expressed as means ± S.D. *p*-value of the difference between groups was measured using Student’s *t* test for comparison. *** *p* < 0.001 was considered statistically significant; (**C**) Migration of HUVEC cells was evaluated by the transwell assay. HUVEC cells incubated with STPC2 (0, 200, 400, and 800 µg/mL, respectively) or STPC2-DeS (800 µg/mL) were seeded in the upper chamber. After 8 h incubation, the migrated cells were stained and photographed; (**D**) The cell viability of HUVECs after STPC2 or STPC2-DeS treatment (0, 200, 400, 800 μg/mL) for 48 h. Each experiment was performed triplicate. Values were present as mean ± S.D.

As elucidated in the previous study [8], STPC2 was demonstrated to be a potential anti-angiogenic agent, but the determinants were still unknown. As discussed in the review [7], the angiogenesis activity of the fucoidans was not only significantly determined by the degree of sulfation, but also highly dependent on their molecular weight, namely fucoidans of high molecular weight were tightly connected with the anti-angiogenic activity, whereas fucoidans of low molecular weight might correlate to their pro-angiogenic capability. To validate the effect of STPC2 structural determinant, we performed the tube formation and transwell assays in vitro. In the tube formation assay (Figure 2A), STPC2 significantly destroyed the ability of HUVEC cells to form capillary tubes compared to the control (0 μg/mL). The inhibition of STPC2 on tube formation was in a dose-dependent manner. A remarkable impairment on the network formation of HUVEC cells was observed in the STPC2 treatment (200 μg/mL) (Figure 2B). In contrast, this disruption of capillary tube formation was not observed in the different doses of STPC2-DeS, even at the high concentration (800 μg/mL) (Figure 2A).

In addition, the cell migration of endothelial cells was also of great importance. To validate the effect of sulfation on the migration, a transwell model was employed. As shown in the Figure 2C, the abilities of STPC2 and its desulfated product were assayed. As shown in the control (0 μg/mL), the cells could freely permeate through the lower chamber. However, after treatment with STPC2 of various dosages for 8 h, the permeated cell number was significantly reduced, and showed in a dose-dependent way. Unexpectedly, the migration of HUVEC was not blocked by the treatment of STPC2-DeS (800 μg/mL). Hence, these in vitro results substantially demonstrated that the anti-angiogenesis of STPC2 was tightly associated with sulfated groups. 

In addition, to identify whether the inhibitory effect of tube formation and migration is the result of cell viability to HUVECs, we applied the MTT assay to detect the cell viability of HUVECs treated with different dosages of STPC2 and STPC2-DeS after incubation for 48 h. As shown in Figure 2D, STPC2 and STPC2-DeS both were observed with no significant effect on the cell viability of HUVECs at these used dosages.

**Figure 3:**
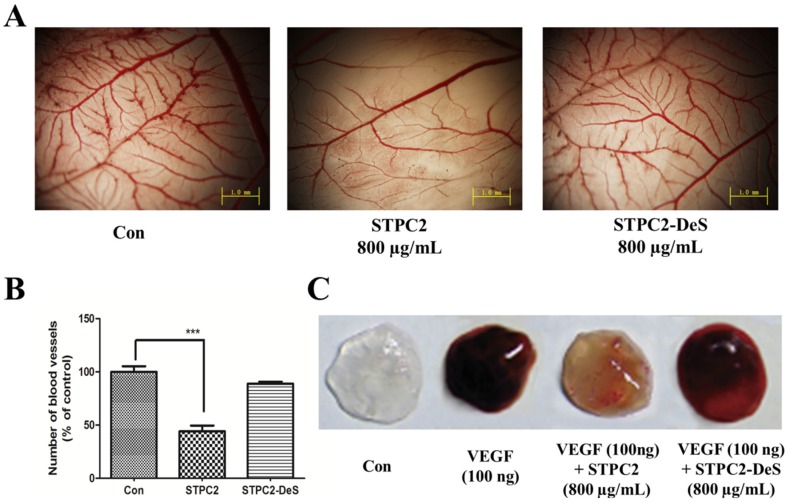
STPC2 inhibited angiogenesis through chorioallantoic membrane (CAM) assay and matrigel plug assay in vivo. (**A**) Filter paper (0.5 cm × 0.5 cm) soaked with saline (control) or saline containing STPC2 (800 µg/mL) or STPC2-DeS (800 µg/mL) was covered on the chorioallantoic membrane. The formed blood vessels on the chick embryo chorioallantoic membrane were photographed after fixation; (**B**) Quantification of the density of newly formed blood vessels. All data were expressed as means ± S.D. *p*-value of the difference between groups was measured using Student’s *t* test for comparison. *** *p* < 0.001 was considered statistically significant; (**C**) Matrigel plug assays were used to evaluate the in vivo inhibition of STPC2 in anti-angiogenic activity. Matrigel with or without VEGF (200 ng/mL) was subcutaneously injected into C57/BL6 mice. Mice were further administrated with STPC2 (10 mg/kg) or STPC2-DeS (10 mg/kg) via tail vein injection every day for two weeks. After the mice were sacrificed, the matrigel plugs were excised and photographed.

To verify the in vivo effect of STPC2 and sulfation contribution, we also conducted the chick chorioallantoic membrane (CAM) assay and matrigel plugs assay. As shown in Figure 3A,B, a significant reduction of microvessel density was observed in the STPC2 treatment, whereas no comparable changes were shown in the STPC2-DeS treatment. In addition, it was reported that VEGF production played a key role in the angiogenesis [12]. Based on this, matrigel treated with VEGF was implanted in mice. Eventually, neovessel formation stimulated by VEGF (200 ng/mL) was notably observed in the matrigel plugs compared to the control (Figure 3C). However, matrigel plugs in the mice that were treated with STPC2 (10 mg/kg) displayed a distinct color fading compared to that treated with VEGF (200 ng/mL) only. On the contrary, the treatment with STPC2-DeS did not show any striking reduction in color. Taken together, it indicated that STPC2 had a potent anti-angiogenesis both in vitro and in vivo, and the presence of sulfated groups highly contributed to the inhibition of angiogenesis.

### 2.3. STPC2 Suppressed the VEGFR2 Activation and Downregulated Its Downstream Signaling

**Figure 4:**
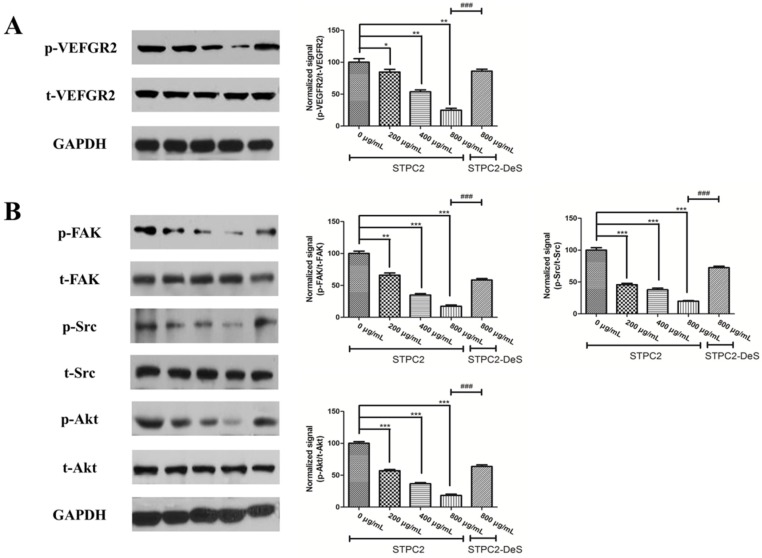
Effect of STPC2 and its desulfated derivative STPC2-DeS on the VEGFR2 and its related signaling molecules in HUVEC cells. HUVEC cells were serum-starved overnight and then incubated with different concentrations of STPC2 (0, 200, 400, and 800 µg/mL) or STPC2-DeS (800 µg/mL) for 1 h. Then proteins were extracted for Western blotting. (**A**) Effect of STPC2 and STPC2-DeS on the phosphorylation of VEGFR2 in HUVEC cells; (**B**) Effect of STPC2 and STPC2-DeS on the phosphorylation of FAK, Src, and Akt in HUVEC cells. All data were expressed as means ± S.D. *p*-value of the difference between groups was measured using Student’s *t* test for comparison. * *p* < 0.05, ** *p* < 0.01, and *** *p* < 0.001 versus STPC2 (0 µg/mL) were considered statistically significant. ^###^ *p* < 0.001 versus STPC2 (800 µg/mL) were considered statistically significant.

VEGF-A could bind to VEGFR2 and trigger its phosphorylation, further activate its various downstream signalings including Src, PKC-Raf-MEK-MAPK cascade, and phosphoinositide 3-kinase (PI3K) associated with its downstream effector Akt to regulate cell proliferation, migration, and tube formation [13]. In the previous study [8], the expressions of VEGF-A and HIF-1α in the endothelial cells were significantly downregulated by the STPC2 treatment. However, the molecular mechanism of anti-angiogenesis mediated by STPC2 has not been demonstrated very well, thus we verified whether the modulation of VEGF-A and HIF-1α was related to the inhibition of VEGFR2 phosphorylation and its subsequent downstream signaling. In this study, the activation of VEGFR2 was observed with a significant downregulation by the treatment of STPC2, while the VEGFR2 expression did not show any distinct alteration. Meanwhile, the treatment of STPC2-DeS failed to trigger the activation of VEGFR2 (Figure 4A). In addition, the effect of STPC2 was accompanied by significant decreases of FAK, and Src phosphorylation (Figure 4B). Moreover, we found that STPC2 could effectively suppress the activation of AKT signaling cascade by repressing the phosphorylated level of AKT (Figure 4B). These results indicated that STPC2 exerted its anti-angiogenesis effect via targeting VEFGR2, and further blockaded its mediated downstream signaling cascade.

### 2.4. STPC2 Strongly Bound to VEGFR2

**Figure 5:**
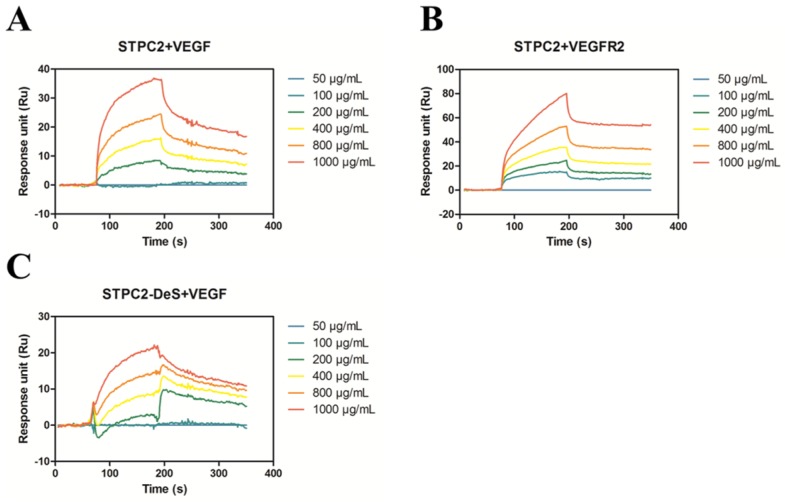
The interaction of native polysaccharide STPC2 with VEGF or VEGFR2, and desulfated derivative STPC2-DeS with VEGF by Biacore 3000. Sensorgrams obtained from the injection of STPC2 or STPC2-DeS over the immobilized VEGF or VEGFR2 surface at the concentrations of 50, 100, 200, 400, 800, 1000 µg/mL. (**A**) Injections of STPC2 over the immobilized VEGF surface at the concentrations of 50, 100, 200, 400, 800, and 1000 µg/mL; (**B**) Injections of STPC2 over the immobilized VEGFR2 surface at the concentrations of 50, 100, 200, 400, 800, and 1000 µg/mL; (**C**) Injections of STPC2-DeS over the immobilized VEGF surface at the concentrations of 50, 100, 200, 400, 800, and 1000 µg/mL.

It was reported that 2-O-sulfate group of hexuronic acid and 6-*O*-sulfation group of *N*-sulfoglucosamine in heparin were necessary for its binding affinity for VEGF165 [14,15]. Fucoidan, an intriguing class of sulfated polysaccharides, had also been regarded as a potential agent for the regulation of angiogenesis based on its special and complicated structural fragments [7]. It was reported that a native fucoidan from *F. vesiculosus* and its over-sulfated derivative could prevent the binding of VEGF165 to its cell surface receptor [16]. We were enlightened from STPC2 specific structural features and its anti-angiogenic molecular basis. Hence, we applied surface plasmon resonance (SPR) to estimate the interaction of VEGF or VEGFR2 with STPC2 by analyzing their affinities. The ratio of *k*_d_ (dissociation rate) to *k*_a_ (association rate) gave the *K*_D_ value, which revealed the affinity of the interactions. In Figure 5 and Table 1, the association rate of STPC2 to VEGF was much higher than that of STPC2 to VEGFR2. In addition, the *K*_D_ for STPC2–VEGF (8.02 × 10^−6^) was less than that for STPC2–VEGFR2 (2.67 × 10^−5^) (Figure 5A,B). The *K*_D_ value indicated that the affinity of STPC2-VEGF is much stronger than that of STPC2–VEGFR2, which indicated STPC2 interfered with the binding of VEGF to VEGFR2, and might modulate the VEGFR2 activation and its downstream signaling. In addition, the K_D_ for STPC2-DeS to VEGF was 2.92 × 10^−5^, which was larger than that of STPC2 to VEGF, indicating the weak affinity of STPC2-DeS to VEGF (Figure 5C).

## 3. Discussion

In this study, we further studied the structure-dependent anti-angiogenesis activity of STPC2 and elucidate the possible underlying mechanism for its regulation on angiogenesis. Firstly, we successfully prepared its desulfated product STPC2-DeS, and analyze the sulfate content. Compared to that of the native polysaccharide, the sulfate content in STPC2-DeS decreased significantly from 27.8% to 2.4%. With the remove of sulfate, its molecular weight was also reduced to 44.5 kD, but the sugar composition in STPC2-DeS did not show any distinct alteration. These results suggested that the desulfation was almost successful and did not make significant structural changes in STPC2-DeS, which guaranteed the reliability of our following experiments. 

Considering the substantial sulfate group in fucoidan and other special chemical groups appearing in its structure, fucoidan has gained more and more interest in recent years. However, the details of structure-dependent activity have not been illustrated very well. Generally, it was considered that high-molecular-weight fucoidans (*M*_w_ > 30 kD) with a high degree of sulfation exhibited a potent anti-angiogenic effect, whereas low-molecular-weight fucoidans (*M*_w_ < 15 kD) promoted angiogenesis [7]. However, the actual situation was more complicated for the structure–activity studies of fucoidans, not only for the difference of molecular weight, but also for the existence of sulfation pattern and degree of sulfation, and other specific hexuronic acid. In order to study the structural determinants in STPC2, we compared the effect of native and desulfated polysaccharides on the tube formation. The results indicated that STPC2 showed a significant inhibition on the capillary network formation in a dose-dependent manner, whereas STPC2-DeS failed to inhibit the tube formation even at the high concentration (800 µg/mL). Thus, it verified our hypothesis that sulfation in STPC2 had a critical role in its regulation on angiogenesis. In addition, in the transwell assay, an obvious interference of endothelial cells infiltrating into the lower well was observed in the STPC2 treatment. In contrast, STPC2-DeS did not show the inhibition of endothelial cell migration in transwell assay. 

Given the visibility, accessibility, and rapid developmental growth of the chorioallantoic membrane (CAM) assay, an in vivo platform for experimentation, this approach has been widely used for the study of vascular development and angiogenesis, tumor growth and metastasis, drug distribution and toxicology [17]. Because of its rapid vascular growth, CAM has been the good model to evaluate the effect of chemical compounds on growing vessels. Many pro- and anti-angiogenic compounds have been tested by quantifying the morphological alteration of the CAM vasculature. To further uncover the structure-dependent anti-angiogenic activity, we performed an in vivo CAM assay. It was found that STPC2 significantly inhibited the blood vessel density in the CAM assay. Meanwhile, we also applied a matrigel plug assay to verify the in vivo effect of STPC2. Matrigel, the basement membrane extract, has been extensively used in in vivo angiogenesis study. Matrigel was regarded to maintain its integrity after subcutaneously injected into mice and form a gel, the vessels could be formed via the stimulation of VEGF. In the matrigel plug assay, STPC2 was found to repress the VEGF-induced neovessel formation. On the contrary, STPC2-DeS did not exhibit the inhibition of new blood vessel growth and formation in the CAM assay and matrigel plug assay. Based on these multiple strategies of angiogenesis studies, these in vitro and in vivo results evidently demonstrated that sulfate groups in STPC2 played a crucial role in its anti-angiogenesis activity.

Angiogenic growth factors such as fibroblast growth factors (FGFs) and vascular endothelial growth factors (VEGFs) have been considered as the targets to inhibit deregulated blood vessel formation [18]. In the previous study [8], STPC2 could downregulate the expression of hypoxia-inducible-factor-1α (HIF-1α) and VEGF, which might impede vascular permeability. However, the molecular mechanism of STPC2 regulating the VEGF expression to affect the anti-angiogenic activity has been illustrated well. 

The high-affinity cognate endothelial receptors for VEGF have also been identified. VEGFR2 is exclusively localized on endothelial cells during embryogenic development and regarded as a key role in endothelial cell differentiation and vasculogenesis [19,20]. These receptors possess intrinsic cytoplasmic enzymatic activity, which can be activated through ligand binding. The binding initiates receptor dimerization and autophosphorylation of tyrosine residues, and further provides docking sites for downstream signaling proteins [21]. In order to uncover the molecular mechanism, we detected the alteration of VEGFR2 and its related downstream signaling molecules. Intriguingly, we found that its phosphorylation was significantly suppressed in the presence of STPC2, but not for STPC2-DeS. In addition, the experimental results also revealed that STPC2 repressed FAK, Src, and Akt activation. It substantially demonstrated that STPC2 exhibited the strong anti-angiogenic activity by the downregulation of VEGFR2 activity, and further modulated the activation of its downstream proteins.

In order to further elucidate the details of interaction between VEGF and VEGFR2 with STPC2, we analyzed the binding affinity of STPC2–VEGF and STPC2–VEGFR2 via SPR assay. In the SPR experiment, the *K*_D_ value of STPC2–VEGF (8.02 × 10^−6^) was approximately three-fold less than that of STPC2–VEGFR2 (2.67 × 10^−5^), which indicated that STPC2 showed a strong binding to VEGF compared to VEGFR2. In addition, the *K*_D_ value of STPC2-DeS to VEGF (2.92 × 10^−5^) was approximately 3.6-fold larger than that of STPC2–VEGF (8.02 × 10^−6^), which indicated the remove of sulfation to some extent reduced the affinity of STPC2 binding to VEGF. By reference to the protein database bank, the α-Fucose, α-_D_-Mannose, *N*-acetyl-_D_-glucosamine and sulfation have been recognized as the ligands of VEGF (DOI: 10.2210/pdb4kzn/pdb). Substantial evidence has shown that β-_D_-Mannose, *N*-acetyl-_D_-glucosamine and sulfation appeared as unique ligands in the crystal structures of VEGFR2 complex [22,23]. Hence, we thought the sulfated groups and α-Fucose in the structure of STPC2 might contribute to the interaction of STPC2 with VEGF. On the contrary, the interaction of STPC2 with its receptor VEGFR2 might function only based on sulfation groups. Thus, we speculated that the interaction of STPC2 with VEGF was more intensive than that of STPC2 with VEGFR2. 

## 4. Material and Methods

### 4.1. Materials

*Sargassum thunbergii* (3.0 kg) was collected from Yantai, Shandong province (China). DEAE-cellulose 52 and Sephacryl S-300 HR were purchased from GE Healthcare Life Sciences (Shanghai, China). Other analytical chemical regents were supplied from Sinopharm (Shanghai, China).

### 4.2. Preparation of Desulfated STPC2

The native polysaccharide, STPC2, was prepared and characterized in the previous publication [8]. Briefly, all the brown alga was extracted from the boiling-water and subjected to precipitation with ethanol. The obtained crude polysaccharide was further purified by CaCl_2_ precipitation and chromatographyed on DEAE-cellulose and Sephacryl S-300 column to yield the homogeneous polysaccharide STPC2. After preliminarily identified the molecular weight, sugar composition, and degree of sulfation of the fresh-fractioned STPC2, the batch of the same physicochemical properties as previously reported was used to prepare its desulfated derivative. Briefly, STPC2 (500 mg) was dissolved in 50 mL of distilled water and loaded on a 732 sulphonic exchange resin column (H^+^) (1.6 cm × 50 cm). The eluate was subjected to neutralization using pyridine and lyophilization. The subsequent process was reference to the previous report [9]. Importantly, the final desulfated product was dialyzed against water with a membrane of small molecular weight cut off (MWCO, 0.1–0.5 kD). The retentate was vacuum-dried to give the desulfated derivative, named as STPC2-DeS.

### 4.3. Physicochemical Properties

High performance gel permeation chromatography (HPGPC) (Agilent, Shanghai, China) was used to determine the homogeneity and molecular weight of polysaccharides. The details were conducted as the previous report [8]. Furthermore, sulfate content was estimated with elemental analysis, carried out on an Elementar Vario EL CUBE machine (Elementar, Germany).

### 4.4. Determination of Sugar Composition

Sugar composition analysis was performed as previously described [8]. Briefly, native or desulfated polysaccharide was hydrolyzed with 2 M TFA at 125 °C for 2 h. After removing extra TFA with methanol, the hydrolyzates were reduced with sodium borohydride for 3 h at room temperature. After neutralized with AcOH and evaporated to dryness, the residue was converted into alditol acetates with Ac_2_O for 1 h at 100 °C. The GC-MS analysis was referenced to the previous report [9]. To quantify the uronic acid content in the structure, both were subjected to carboxyl-reduction referenced by the publication [24]. The carboxyl reduced products were hydrolyzed and analyzed again as described above.

### 4.5. Infrared Spectroscopy (IR)

The IR spectrum was analyzed on a Nicolet 6700 FT-IR spectrometer (Thermo Scientific, Waltham, MA, USA). Desulfated polysaccharide (STPC2-DeS, 2 mg) was ground with KBr and then pressed into pellets, and further scanned from 4000 to 400 cm^−1^.

### 4.6. Cell Lines and Culture Conditions

Human umbilical vein vascular endothelial cells (HUVECs) were obtained from the Cell Bank of the Chinese Academy of Sciences, and cultured in RPMI 1640 medium (Gibco BRL, Gaithersburg, MA, USA) supplemented with 10% FBS, penicillin (50 U/mL), and streptomycin (50 µg/mL). All the cells were incubated at 37 °C with 5% CO_2_ in a humidified atmosphere.

### 4.7. Tube Formation Assay of HUVEC

The effect of STPC2 and STPC2-DeS on the formation of capillary tube-like structures was evaluated in a matrigel-based assay as previously reported [25]. Briefly, a 96-well plate pre-coated with 50 μL matrigel each well was solidified at 37 °C for 1 h. Human umbilical vein vascular endothelial cells (HUVECs) were trypsinized, and supplemented with RPMI 1640 only or containing different doses of STPC2 or STPC2-DeS (0, 200, 400, and 800 µg/mL). The plate was incubated again for additional 24 h. The enclosed capillary network was photographed by an Olympus IX51 digital camera (Tokyo, Japan). Quantification of the inhibition of tube formation was analyzed by Image J software (NIH, Bethesda, MD, USA).

### 4.8. Cell Migration by Transwell Assay

The migration of HUVECs was evaluated by a transwell assay in a 24-well, 8-μm-pore size transwell plate (Costar, Cambridge, MA, USA). HUVECs (2 × 10^5^ cells/well) were seeded in the upper chamber in 100 μL of serum free medium containing different concentrations of STPC2 (0, 200, 400, and 800 µg/mL) or STPC2-DeS (800 µg/mL). The lower chamber was maintained with same medium including 10% FBS. After incubated for 8 h, the migrated cells were stained with 0.1% crystal violet. While the non-migrated cells in the upper surface of the membrane were removed using a cotton swab. Migrated cells were then photographed by a microscope (Olympus BX510, Tokyo, Japan).

### 4.9. Chick Embryo Chorioallantoic Membrane Assay

The chorioallantoic membrane (CAM) assay, an in vivo platform for the study of vascular development and angiogenesis was conducted as previously described [17]. Briefly, fertilized chick eggs were incubated at 37 °C in a humid atmosphere. After one week, the eggshell was cracked and peeled away over the airspace to open a small window. A 0.5 cm × 0.5 cm sheet of filter paper soaked with saline (control) or saline containing STPC2 (800 µg/mL) or STPC2-DeS (800 µg/mL) was covered on the chorioallantoic membrane. The eggs were sealed tightly and maintained in the incubator for another 48 h. Finally, the CAMs were taken to photograph with a microscope (Olympus BX510, Japan) after fixation (methanol:acetone = 1:1).

### 4.10. Matrigel Plug Assay

The experiment was carried out as described previously [26]. C57/BL6 mice were got from Shanghai Laboratory Animal Center of the Chinese Academy of Sciences. All the protocols were carried out in accordance with the Guide for the Care and approved by the Animal Ethics Committee of Hospital. Briefly, we subcutaneously injected 0.5 mL of matrigel containing 100 ng VEGF into the ventral area of mice (*n* = 5 per group). Control group mice were only injected with matrigel, whereas the experimental group mice were administrated with STPC2 (10 mg/kg) or STPC2-DeS (10 mg/kg) via tail vein injection every day for two weeks. After the mice were sacrificed, the matrigel plugs were excised and photographed.

### 4.11. Cell Viability Assay of STPC2 and STPC2-DeS

The cytotoxicity of STPC2 and STPC2-Des was evaluated on the HUVEC cells by the MTT assay. HUVEC cells (5 × 10^3^ cells/ well) were seeded on 96-well plates in triplicate for 24 h. The medium was exchanged with different concentrations (0 µg/mL, 200 µg/mL, 400 µg/mL, 800 µg/mL) of STPC2 and STPC2-DeS, respectively, dissolved in corresponding medium for another 48 h. Subsequently, 20 µL of 5 mg/mL MTT was added into each well and incubated for 4 h at 37 °C. The medium was discarded and the formazan crystal was dissolved in 200 µL of DMSO. The absorption at 490 nm was recorded to calculate the cell viability ratio.

### 4.12. Western Blotting

HUVEC cells were serum-starved for overnight and then incubated with different concentrations of STPC2 (0, 200, 400, and 800 µg/mL) or STPC2-DeS (800 µg/mL) for 1 h. After that, the cells were lysed with RIPA buffer, according to the manufacturer’s instruction (Cell Signaling Technologies, Shanghai, China). The concentrations of proteins were determined by BCA protein assay kit (Beyotime, Haimen, Jiangsu, China). Equivalent amounts of proteins from different treatments were electrophoresised on SDS-PAGE gels and transferred to a polyvinylidene difluoride (PVDF) membrane (Bio-Rad, Hercules, CA, USA). Blots were performed with epitope-specific primary and secondary antibodies. Anti-VEGFR2 antibody, anti-VEGFR2 (phospho Y1054/1059) antibody, anti-FAK anbibody, anti-FAK (phospho Y397) antibody, anti-Src antibody, and anti-Src (phospho Y418) antibody were got from Abcam. Anti-Akt and phospho-Akt (Thr308) antibodies were purchased from Cell signaling technology. Finally, enhanced chemiluminescence (ECL) reagent was applied for signal detection. The data were quantified and normalized using image J software.

### 4.13. Surface Plasmon Resonance Analysis

To further uncover the details of interference of STPC2 with the interaction between VEGF and VEGFR2, surface plasmon resonance (SPR) analysis was used to study their respective affinities. The SPR experiment was carried out as previous report [27]. Briefly, proteins (VEGF or VEGFR2) were immobilized on a CM5 sensor chip by the amine coupling method [28]. For interaction determination, different concentrations of STPC2 or STPC2-DeS (50, 100, 200, 400, 800, and 1000 µg/mL) were injected into to the chips. All procedures were performed in HBS-EP (0.15 M NaCl, 0.01 M HEPES, 3 mM EDTA and 0.005% surfactant P20, pH 7.4) running buffer. Eventually, kinetic parameters were analyzed by BIACORE T200 Evaluation Software Version 1.0 (GE Healthcare, Uppsala, Sweden).

### 4.14. Statistical Analysis

All data were expressed as means ± S.D. *p*-value of the difference between groups was measured using Student’s *t* test for pair comparison or one-way ANOVA for multiple comparisons. * *p* < 0.05, ** *p* < 0.01, and *** *p* < 0.001 were considered statistically significant.

## 5. Conclusions

Taken together, in this study, we demonstrated that STPC2 might show a potent anti-angiogenic activity through binding to VEGF via the structural α-Fucose and sulfation to impair the interaction between VEGF and VEGFR2, and thus further affect the downstream signaling molecules related to VEGFR2.

## Figures and Tables

**Table 1:** Association (*k*_a_) and dissociation (*k*_d_) rate constants and apparent equilibrium dissociation (*K*_D_) constants of STPC2 interactions with VEGF or VEGFR2, and STPC2-DeS interactions with VEGF.

Samples	*k*_a_ (1/Ms)	*k*_d_ (1/s)	*K*_D_ (M)
STPC2 + VEGF	526	4.22 × 10^−3^	8.02 × 10^−6^
STPC2 + VEGFR2	249	6.66 × 10^−3^	2.67 × 10^−5^
STPC2-DeS + VEGF	140	4.10 × 10^−3^	2.92 × 10^−5^
